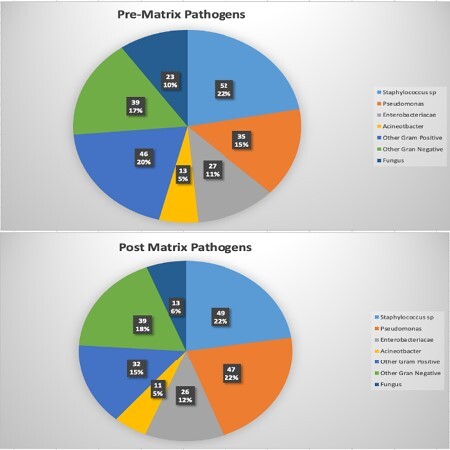# 604 Single Center 4-year Review of Synthetic Polyurethane Matrix Use in Burns and Other Complex Wounds

**DOI:** 10.1093/jbcr/irae036.238

**Published:** 2024-04-17

**Authors:** Muntazim Mukit, Payton Grande, David M Hill, Sai R Velamuri, Mahmoud Hassouba, Xiangxia Liu

**Affiliations:** University of Tennessee Health Science Center, Memphis, TN; Regional One Health, Memphis, TN; Firefighters Burn Center, Regional One Health, Memphis, TN; University of Tennessee Health Science Center, Memphis, TN; Regional One Health, Memphis, TN; Firefighters Burn Center, Regional One Health, Memphis, TN; University of Tennessee Health Science Center, Memphis, TN; Regional One Health, Memphis, TN; Firefighters Burn Center, Regional One Health, Memphis, TN; University of Tennessee Health Science Center, Memphis, TN; Regional One Health, Memphis, TN; Firefighters Burn Center, Regional One Health, Memphis, TN; University of Tennessee Health Science Center, Memphis, TN; Regional One Health, Memphis, TN; Firefighters Burn Center, Regional One Health, Memphis, TN; University of Tennessee Health Science Center, Memphis, TN; Regional One Health, Memphis, TN; Firefighters Burn Center, Regional One Health, Memphis, TN

## Abstract

**Introduction:**

As not all wounds are amenable to immediate skin grafting, the search for the ideal skin substitute is ever present. The ideal skin substitute would be resistant to infection, inexpensive, readily available, quick to integrate and applicable to a variety of wounds. Here we describe the use of a synthetic polyurethane matrix in the setting of burns and other complex wounds in the largest case series to date.

**Methods:**

A retrospective review was conducted at a verified, regional burn center. Dual IRB approval was obtained. All patients greater than 18 years of age who received this matrix between January 2019 and July 2023 were included. Data collected included: age, sex, wound etiology, social and medical history, presence of exposed critical structures, presence of infection, length of stay, time to matrix implantation, time to skin graft, percent of matrix survival, percent skin graft survival and disposition.

**Results:**

A total of 182 patients with 250 wounds were included in this study. The average age was 50.5 ± 18.7 years and 61% were male. Thirty-seven percent were smokers, 23.6% had diabetes and 5.5% had peripheral vascular disease. The majority were acute burn wounds (60%), followed by, trauma (24.4%), chronic wounds (5.6%), infection (4%) and donor sites (2%). Exposed structures included fat (54%), muscle (30%), bone (16%) and tendon (14.4%). Infection was present in 143 (57.2%) of cases: 98 (39.2%) pre-application and 89 (35.6%) post application. The incidence of new infection post-application was 25.6%. The pathogens are detailed in figure 1; several infections were polymicrobial. Eighteen patients (10%) died. Median length of stay was 27 (13.3, 64) days. Median time to matrix implantation was 10 (6, 17) days. And median time from matrix placement to skin grafting was 35 (24.8, 42) days. Where documented, there were 162 wounds (83.5%) with > 95% matrix survival and 136 wounds (82.4%) with > 95% skin graft survival. The patient dispositions are detailed in table 2.

**Conclusions:**

We describe to date the largest clinical series utilizing a synthetic polyurethane matrix. This matrix was successfully used in a large number of patients with burns, trauma and chronic wounds. Despite the presence of active smoking, diabetes or infection, there was greater than 95% matrix and skin graft survival in more than 80% of patients. This study demonstrates the robustness of this skin substitute to achieve successful reconstruction even in the setting of adverse patient or wound characteristics.

**Applicability of Research to Practice:**

This study highlights the utility of using a synthetic polyurethane matrix to cover full thickness, difficult to reconstruct wounds secondary to trauma or other injuries. This matrix is resistant to infection and can allow for successful coverage of wounds even in patients who are smokers or have diabetes.